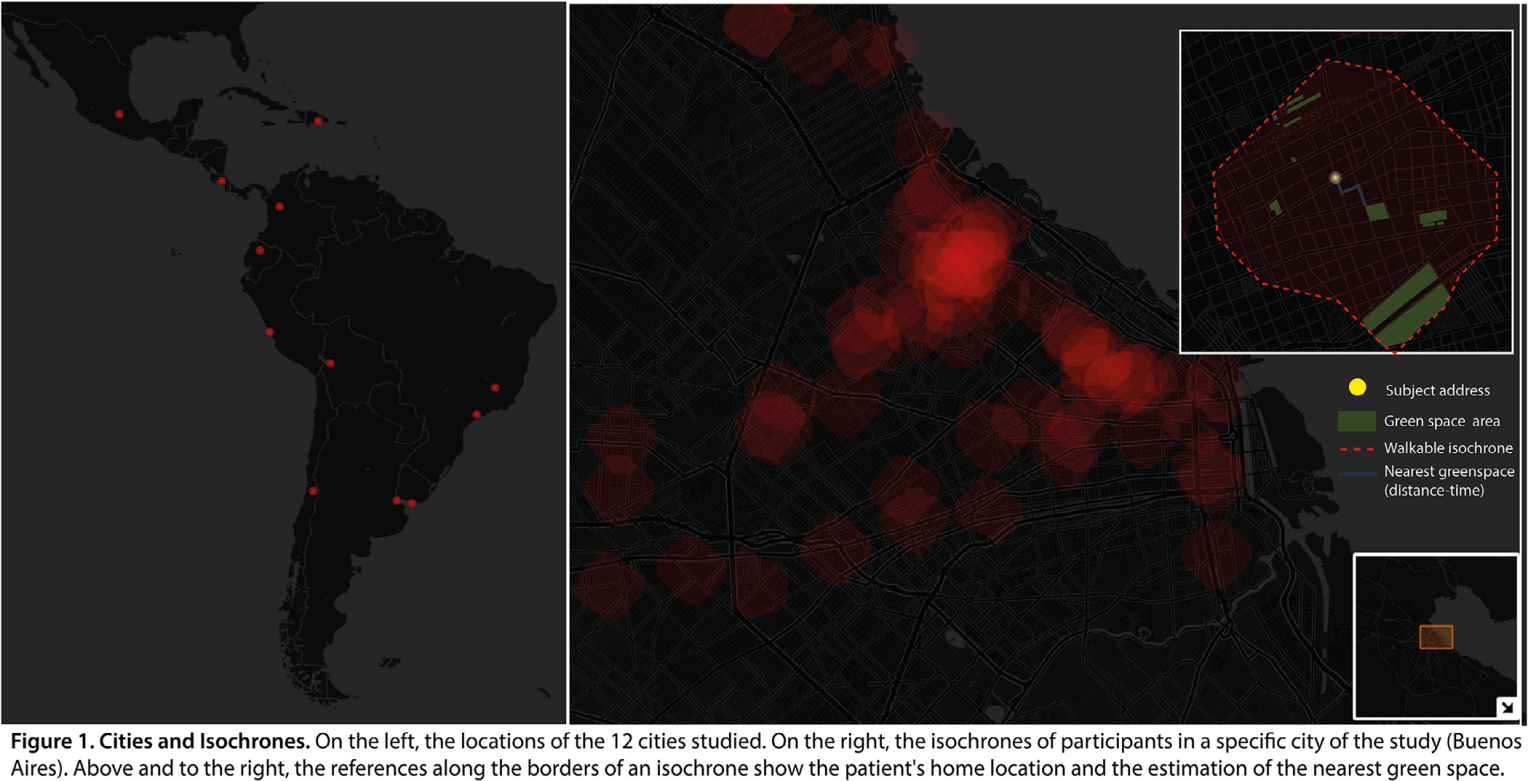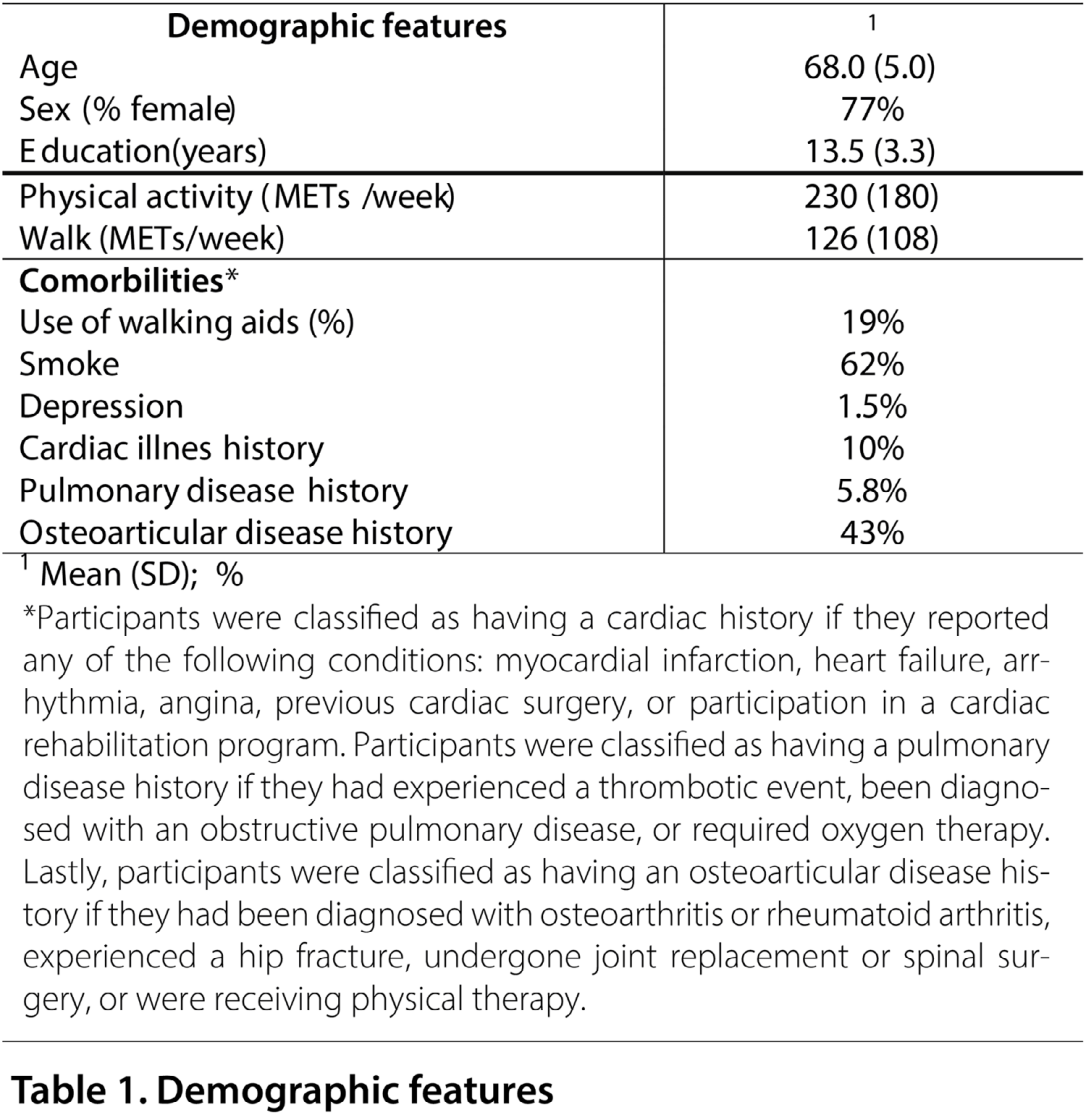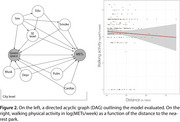# Integrating city design into lifestyle trials: urban greenness shapes physical activity levels across 12 cities in the LatAm FINGERS trial

**DOI:** 10.1002/alz70860_107127

**Published:** 2025-12-23

**Authors:** Ignacio Spiousas, María Florencia Coldeira, Claudia Kimie Suemoto, Ana Y Baena, Rosa Maria Salinas‐Contreras, Paulo Caramelli, Sonia Brucki, Ricardo Nitrini, Ana Luisa Sosa, Luc¡a Crivelli, Gustavo Sevlever, Nilton Custodio, Daisy M Acosta, Ana Charamelo, Jorge Leon Salas, Carolina Delgado, María Isabel Cusicanqui, Ivonne Z. Jimenez Velazquez, David Aguillon, Lissette Duque, Ismael Luis Calandri, LatAm‐FINGERS consortium

**Affiliations:** ^1^ Laboratorio Interdisciplinario del Tiempo (LITERA), Universidad de San Andrés/CONICET, Buenos Aires, Buenos Aires, Argentina; ^2^ Physiopathology in Aging Laboratory (LIM‐22), Department of Internal Medicine, University of São Paulo Medical School, São Paulo, São Paulo, Brazil; ^3^ Grupo de Neurociencias de Antioquia, Facultad de Medicina, Universidad de Antioquia, Medellín, Colombia; ^4^ Dementias Laboratory, National Institute of Neurology and Neurosurgery, Mexico City, DF, Mexico; ^5^ Universidade Federal de Minas Gerais, Belo Horizonte, MG, Brazil; ^6^ University of São Paulo Medical School, São Paulo, Brazil; ^7^ Hospital das Clínicas da Faculdade de Medicina da Universidade de São Paulo, São Paulo, São Paulo, Brazil; ^8^ Instituto Nacional de Neurología y Neurocirugía, Mexico City, DF, Mexico; ^9^ Fleni, Buenos Aires, Argentina; ^10^ Fleni, CABA, Buenos Aires, Argentina; ^11^ Unidad de Investigación, Instituto Peruano de Neurociencias, Lima, Lima, Peru; ^12^ Asociación Dominicana de Alzheimer y otras demencias, Santo Domingo, Santo Domingo, Dominican Republic; ^13^ Hospital Británico, Clínica de Memoria, Montevideo, Montevideo, Uruguay; ^14^ LatamFINGERS, San Jose, Costa Rica; ^15^ Universidad de Chile, Santiago, Santiago, Chile; ^16^ Centro Neurológico Mente Activa, La Paz, Bolivia (Plurinational State of); ^17^ Centro de Investigación en Geriatría, Recinto de Ciencias Médicas, Universidad de Puerto Rico, San Juan, PR, USA; ^18^ Neurosciences Group of Antioquia, University of Antioquia, Medellín, Colombia; ^19^ Neuromedicenter, Quito, Ecuador; ^20^ Alzheimer center, VUMC, Amsterdam, Netherlands; ^21^ Fleni, Buenos Aires, Buenos Aires, Argentina

## Abstract

**Background:**

LatAm FINGERS is a feasibility trial aimed at preventing cognitive decline in individuals at risk through lifestyle changes in Latin America, with its objectives aligned with the RE‐AIM (Reach, Effectiveness, Adoption and Implementation) framework. An essential component of this framework is adoption, which emerges from the interaction between participants, the trial procedures, and their environment. Given that the intervention focuses on lifestyle modifications, it is crucial to consider an often‐overlooked factor: the city where participants live and its influence on behavior. A clear example is the relationship between physical activity and the availability of spaces that support it. Urban greenness refers to the collection of green spaces within a city. We aimed to examine how accessibility to green spaces influences the amount of physical activity performed by participants before starting the LatAm‐FINGERS trial.

**Method:**

We geolocated the residences of 1,800 screened individuals from 12 Latin American cities. Using publicly available data, we estimated isochrones, or areas reachable within a 15‐minute walk (3.4 km/h), along city pathways. As a measure of greenness, we calculated the surface area of public green spaces within each isochrone and the distance to the nearest green space. Physical activity was assessed using the International Physical Activity Questionnaire (IPAQ) and converted into metabolic equivalent of task (MET) units. A generalized linear mixed‐effects model assessed whether weekly physical activity levels (in METs/week) were associated with walkable green space availability. The model was adjusted for socioeconomic status, education, sex, mobility assistance needs, depression, smoking, and co‐morbid conditions.

**Result:**

Walking was the most common physical activity, with weekly METs averaging 126.5 (SD=108.4), representing 70.2% (SD=10.8%) of total physical activity. While the total green space area had a minimal effect on walking (β=−0.0004, 95%CI=‐0.0017, 0.0001), distance to the nearest green space influenced walking levels (β=‐0.0440, 95%CI=‐0.1874, 0.0982). For each block farther a participant's home was from a green space, weekly walking METs decreased by 3%.

**Conclusion:**

These results highlight the importance of green spaces in promoting physical activity. Future steps will explore whether urban greenness also influences intervention adherence to the physical activity program and other measures of wellbeing.